# The median effective dose of ciprofol combined with sufentanil in suppressing the laryngeal mask airway insertion response in both young and older adult patients

**DOI:** 10.1186/s12871-024-02855-5

**Published:** 2024-12-19

**Authors:** Xuelei Zhou, Li Zhao, Wei Mao, Linlin Chen, Xianchun Liu, Linji Li

**Affiliations:** Department of Anesthesiology, The Second Clinical Medical College, North Sichuan Medical College, Nanchong Central Hospital, Nanchong, China

**Keywords:** Ciprofol, Median effective dose, Laryngeal mask airway, Young and older adult patients, Sequential method

## Abstract

**Background:**

Ciprofol, a novel intravenous anesthetic, exhibits similar sedation mechanisms and pharmacokinetic properties to propofol. However, ciprofol demonstrates greater potency and is associated with reduced injection pain compared to propofol. Given the varying sensitivities to anesthetic agents across different age groups, this study aims to determine the median effective dose (ED_50_) of ciprofol required to suppress the laryngeal mask airway (LMA) insertion response in both young and older adult patients, as well as to assess its potential adverse reactions.

**Methods:**

In this study, 46 patients scheduled for surgery under general anesthesia with LMA insertion were recruited. Upon entering the operating room, patients were intravenously administered ciprofol (0.4 mg·kg^− 1^) and sufentanil (0.3 µg·kg^− 1^), followed by LMA insertion after three minutes. To derive robust confidence intervals for both ED_50_ and ED_95_, we performed an analysis using a logistic regression model combined with bootstrap resampling.

**Results:**

In the young adult group, the ED_50_ and ED_95_ of ciprofol for suppressing the LMA insertion response were 0.38 mg·kg^− 1^ (95% CI, 0.35–0.41) and 0.46 mg·kg^− 1^ (95%CI, 0.40–0.56), respectively. In the older adult group, the respective ED_50_ and ED_95_ were 0.29 mg·kg^− 1^ (95% CI, 0.26–0.32) and 0.37 mg·kg^− 1^ (95% CI, 0.30–0.78). Regarding adverse reactions, although there were differences in the incidence of injection pain, hypotension, and bradycardia between the young and older groups, no statistically significant differences were observed between the two groups.

**Conclusion:**

In this study, significant differences were observed in the ED_50_ of ciprofol for suppressing the LMA insertion response between young and older adult patients. The ED_50_ of ciprofol for young adult patients was 0.38 mg·kg^− 1^ (95% CI, 0.35–0.41), while for older adult patients it was0.29 mg·kg^− 1^ (95% CI, 0.26–0.32).

**Trial registration:**

This study was registered on February 17, 2024, with the China Clinical Trial Registration Center (www.chictr.org.cn; Registration Number: ChiCTR2400080891).

**Supplementary Information:**

The online version contains supplementary material available at 10.1186/s12871-024-02855-5.

## Background

With the global trend of aging, the frequency of surgeries among older adults is rapidly increasing [1, 2]. Compared to younger individuals, older adults naturally experience a decline in physiological functions, leading to reduced tolerance to stress responses and heightened sensitivity to anesthetic drugs [3–6]. Therefore, developing appropriate perioperative pharmacological strategies for both young and older patients is important [7].

The Laryngeal Mask Airway (LMA) is commonly used in airway management due to its minimally invasive nature [8–11]. However, LMA insertion may still provoke stress responses and lead to cardiovascular instability [12, 13]. Reasonable selection of anesthetic dosage to suppress these reactions can help improve patient safety.

Propofol is a widely used intravenous anesthetic in clinical settings, extensively applied in anesthetic induction, maintenance, and outpatient surgery anesthesia [14, 15]. However, propofol often induces adverse reactions such as injection pain, hypotension, bradycardia, and respiratory depression [16]. In this context, ciprofol, a novel intravenous anesthetic, shares similar sedation mechanisms and pharmacokinetic properties with propofol, but it demonstrates greater potency and a lower incidence of adverse reactions [17–21]. Considering the varying sensitivity of different age groups to anesthetic drugs [5], this study seeks to investigate the median effective dose (ED50) of ciprofol combined with sufentanil in suppressing the LMA insertion response in both young and older patients, while also observing its potential adverse reactions.

## Materials and methods

### Experimental design and calculations

Patients who underwent elective general anesthesia with laryngeal mask airway (LMA) insertion at Nanchong Central Hospital, Nanchong City, Sichuan Province, between February and March 2024, were selected as the subjects of this study. Based on age differences, patients were divided into two groups to explore the potential impact of age on ciprofol dosage. The Dixon’s up-and-down method was employed to accurately determine the ED_50_. Starting from a dose nearest to the anticipated ED_50_, the Dixon’s up-and-down method involves adjusting the dosage level up or down based on the previous subject’s response, with the ED_50_ calculated after six crossover points [22]. In terms of sample size, based on the Dixon up-and-down method to estimate the ED50, each group typically requires 20 to 30 participants for reliable estimation. Given that the study involves two groups, the study plans to recruit between 40 and 60 participants.

### Inclusion criteria

Patients undergoing LMA general anesthesia surgeries were recruited, including two age groups: 18–44 years and 60–79 years. Patients needed to be classified between American Society of Anesthesiologists (ASA) grades I to III. The study was open to all genders, with participants required to voluntarily participate and sign an informed consent form.

### Exclusion criteria

Baseline measurements: systolic blood pressure over 160 mmHg or diastolic blood pressure over 100 mmHg; heart rate lower than 60 bpm or higher than 100 bpm; acute respiratory infection, acute exacerbation of chronic obstructive pulmonary disease (COPD), or poorly controlled asthma; mental disorders including schizophrenia, depression, cognitive impairments, etc.; allergy to the study drugs; history of benzodiazepine medication in the past three months; pregnant or lactating women; patients with anticipated difficult airways; body mass index less than 19 kg·m^− 2^ or greater than 28 kg·m^− 2^; and severe comorbidities (including but not limited to renal failure, hepatic failure, cerebrovascular accidents, heart failure, myocardial infarction, etc.)

### Withdrawal criteria

Patients will be withdrawn from the primary analysis in cases of unexpected difficult airways or severe adverse reactions, such as allergies. However, all withdrawn cases will be documented in detail, particularly any adverse events, to ensure comprehensive reporting and patient safety monitoring.

## Blinding and grouping

We selected eligible patients undergoing LMA general anesthesia surgeries and divided them into two groups based on their age: the Youth group (Q group, aged 18–44) and the Older Adult group (L group, aged 60–79).

### Study execution

The study coordinator was responsible for implementing the research protocol. Anesthetists were provided with ciprofol and sufentanil in syringes (ciprofol in 20 ml syringes, sufentanil in 10 ml syringes) that appeared identical but contained varying concentrations; Data Collection and Processing: Data collection was conducted by a researcher unaware of the drug dosages to ensure objectivity. The anesthetist implementing the research protocol and the researcher responsible for data collection did not share any information regarding patient drug allocation, maintaining the blindness of the study; Statistical Analysis: All collected data will be handed over to a designated researcher for independent statistical analysis.

## Anesthesia protocol

Pre-anesthesia Preparation: Patients are required to adhere to strict dietary restrictions before surgery, including fasting for at least 8 h and abstaining from drinking for 6 h. Prior to surgery, routine monitoring of vital signs is conducted, including non-invasive blood pressure(BP), heart rate(HR), pulse oximetry (SpO_2_), and bispectral index (BIS). To obtain accurate baseline vital sign data, vital signs are measured continuously three times within 10 min of initial measurement, and the average of these three results is taken as the baseline value (T0). The patient is positioned supine and administered oxygen at 3 L/min via a face mask.

Anesthetic Induction and LMA Insertion: Sufentanil (0.3 µg·kg^− 1^) and ciprofol are sequentially administered intravenously at a slow rate, followed by assisted ventilation for three minutes. Once the Modified Observer’s Alertness/Sedation scale (MOAA/S) score reaches 0, an experienced anesthetist performs the LMA insertion, choosing the size based on the patient’s weight (3# for 30–50 kg, 4# for 50–70 kg) and lubricating it with lidocaine gel. Upon successful insertion, the patient is either maintained on spontaneous respiration or commenced on mechanical ventilation as per clinical requirements. The initial dose of ciprofol is set at 0.4 mg·kg^− 1^, and the dosage for the next patient is determined based on the presence or absence of a laryngeal mask insertion response. The laryngeal mask insertion response is defined as a score greater than 4 on a six-category scale (a-f) [23]. This is calculated based on occurrences of swallowing, coughing or gagging, head or body movement, and laryngospasm during insertion:

- Swallowing grading nil = 1, slight = 3, gross = 3.
- Coughing and gagging grading nil = 1, slight = 3, gross = 3.
- Head or body movement grading nil = 1, slight = 3, gross = 3.
- Laryngospasm grading nil = 1, partial = 2, total = 3。.

Due to the uniqueness of the upper airway anatomy: the degree of mouth opening and the ease of insertion were excluded from consideration [24]. If no LMA insertion response occurs, the ciprofol dose for the next patient is reduced by 0.05 mg·kg^− 1^. If a response is observed, the dose is increased by 0.05 mg·kg^− 1^ for the subsequent patient. Patients who experienced LMA insertion response were administered intravenous ciprofol 0.1–0.2 mg/kg to deepen anesthesia.

Anesthesia Maintenance and Recovery: Three minutes after the LMA insertion, anesthesia is maintained with sevoflurane. Intravenous administration of cisatracurium (0.15 mg·kg^− 1^) is used to provide adequate muscle relaxation for the surgery, and intermittent intravenous sufentanil is used for pain management. After laryngeal mask removal at the end of the surgery, the patient is transferred to the Post Anesthesia Care Unit (PACU).

Adverse Reactions and Management: In case of a laryngeal mask insertion reaction, ciprofol 1 mg·kg^− 1^ may be administered to deepen anesthesia as needed. If laryngeal mask insertion is unsuccessful or an unexpected difficult airway occurs, intravenous ciprofol 0.4 mg·kg^− 1^ and cisatracurium 0.2 mg·kg^− 1^ are administered to deepen anesthesia before proceeding with endotracheal intubation. Blood Pressure Increase: If SBP/DBP increases by more than 30% above the baseline, anesthesia may be deepened or urapidil administered as appropriate. Blood Pressure Decrease: If SBP/DBP decreases by more than 30% from the baseline or blood pressure is less than 85/50 mmHg, an appropriate dose of ephedrine or norepinephrine is administered. Bradycardia: Atropine 0.3 mg is given if HR < 50 bpm. Tachycardia: Esmolol is administered as appropriate if HR > 100 bpm.

## Outcome measures

### Primary outcome

Observing whether a LMA insertion reaction occurs during the insertion process.

### Secondary outcomes

Vital signs and adverse reactions during anesthetic induction; Recording of HR, SpO2, systolic blood pressure(SBP), diastolic blood pressure(DBP), mean arterial pressure(MAP), and BIS values at T0 (the average of three measurements taken within 10 min of entering the room), T1 (prior to laryngeal mask insertion), and T2 (3 min after laryngeal mask insertion); recording of the highest HR, SDP, DBP, MAP, and SpO2 at T3 (within 3 min after laryngeal mask insertion); recording the time taken to complete laryngeal mask insertion; recording the number of adverse reactions within 3 min after induction and laryngeal mask insertion, including: increased blood pressure, decreased blood pressure, bradycardia, tachycardia, reduced oxygen saturation (< 90), injection pain, and other adverse reactions.

### Statistical analysis

The data were processed using SPSS software version 26.0 and R software version 4.4.1. The normality of data was assessed using the Shapiro-Wilk test. Data following a normal distribution are presented as mean ± standard deviation (X ± SD), and non-normally distributed data are presented as median (interquartile range). The t-test was used for parametric comparisons, and the Mann-Whitney U test for analyzing non-parametric tests between two independent samples. Categorical variables were compared using the chi-square test. Repeated measures analysis of variance (rmANOVA) was used to analyze changes in MAP and HR over time, with Bonferroni correction applied for multiple pairwise post-hoc comparisons. We initially planned to estimate the ED50 of ciprofol for suppressing the LMA insertion response using Dixon’s up-and-down method and probit regression analysis. To improve the robustness of the ED50 and ED95 estimates and their confidence intervals, we applied a logistic regression model combined with bootstrap resampling. The logistic regression provided estimates based on the observed dose-response relationship, while bootstrap resampling avoided distributional assumptions, resulting in more reliable confidence intervals. A statistical difference between the two groups was indicated if there was no overlap in the ED_50_ (95% CI) between them [25]. A p-value < 0.05 was considered statistically significant.

## Results

### Primary outcome

**Fig. 1:**
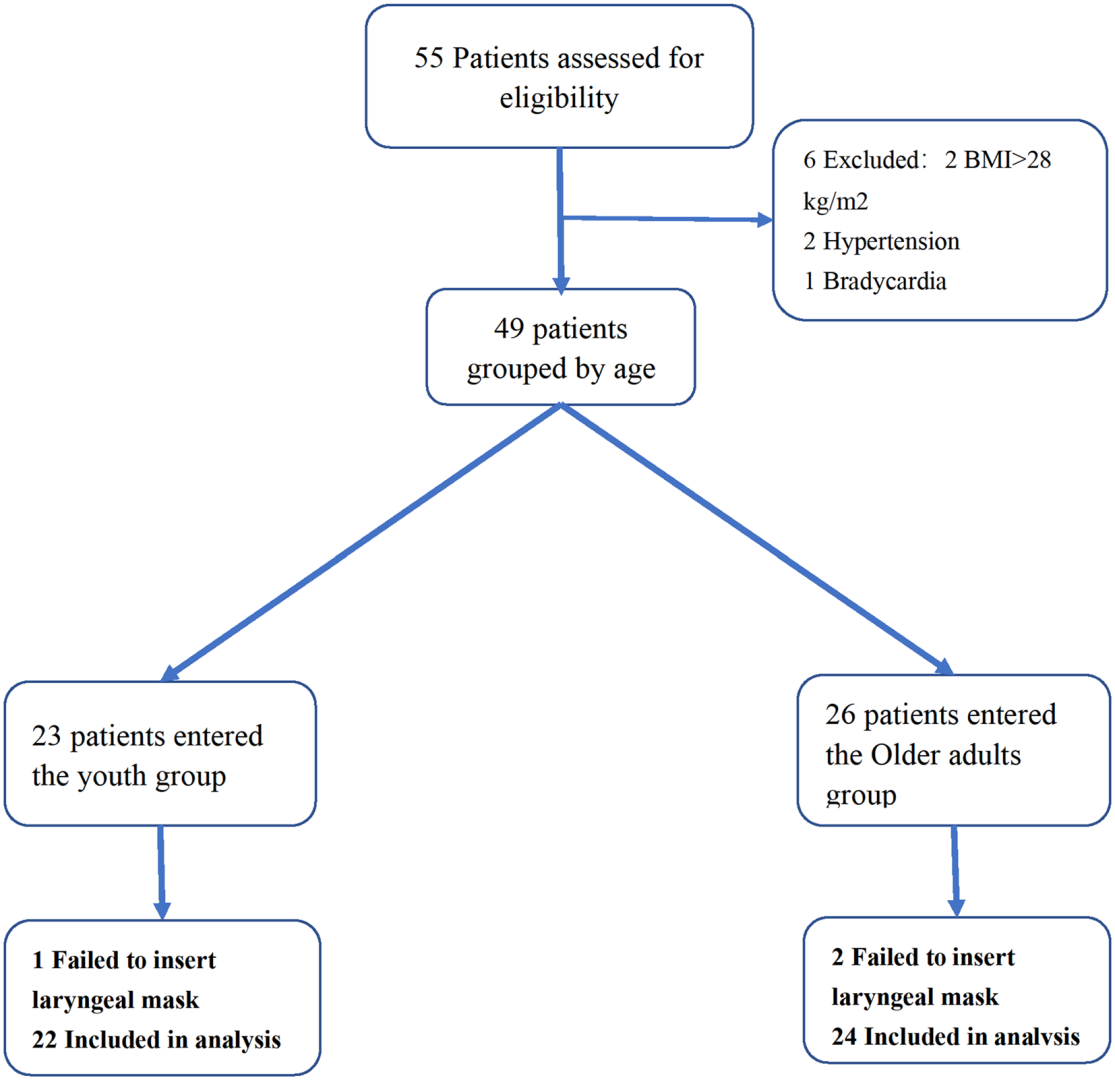
flowchart

In this study, 55 patients were initially screened. During further screening, 2 patients were excluded due to a BMI greater than 28, 2 due to hypertension, and 1 each due to bradycardia and schizophrenia, resulting in a total of 49 patients being included. After age grouping, there were 23 patients in the youth group and 26 in the older adult group. Due to inadequate sealing and subsequent insertion failure, one patient from the youth group and two from the older adult group withdrew from the study and underwent tracheal intubation. In the end, 46 patients were included in this study (Fig. 1). There were no statistically significant differences between the two groups in terms of weight, height, and BMI. However, statistically significant differences were found in ASA grades and gender (Fig. 1). There were no statistically significant differences between the two groups in terms of weight, height, and BMI. However, statistically significant differences were found in ASA grades and gender (Table 1).

**Table 1:** Demographic characteristics of the two groups

	Youth group (*n* = 22)	Older adults group (*n* = 24)	*p*-value
Age (years)	34.41 ± 6.24	65.50 ± 5.02	
Weight (kg)	59.32 ± 12.02	59.55 ± 7.02	0.935
Height (cm)	159.77 ± 9.21	161.63 ± 7.00	0.800
BMI (kg/m^2^)	23.03 ± 2.65	22.78 ± 2.29	0.729
Gender (M/F)	6/16	8/16	0.018
ASA (I/II/III)	7/14/1	0/20/4	0.016

Data are presented as the mean ± SD or the number; M Male F Female; BMI Body mass index; ASA American Society of Anesthesiologists; P-value: Independent Samples t-Test/Chi-Square Test

**Fig. 2:**
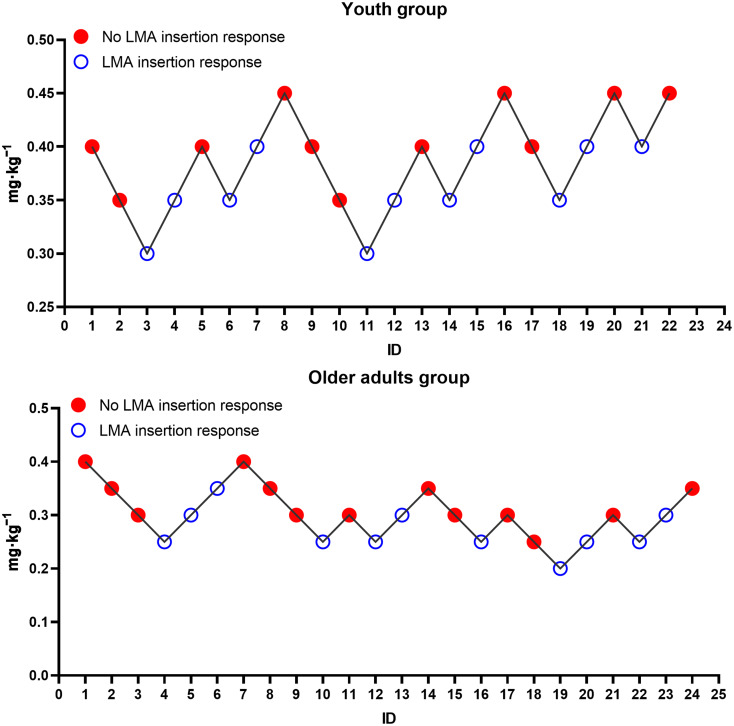
Dixon up-and-down plots; Red indicates no LMA insertion response; Blue indicates the presence of LMA insertion response

**Fig. 3:**
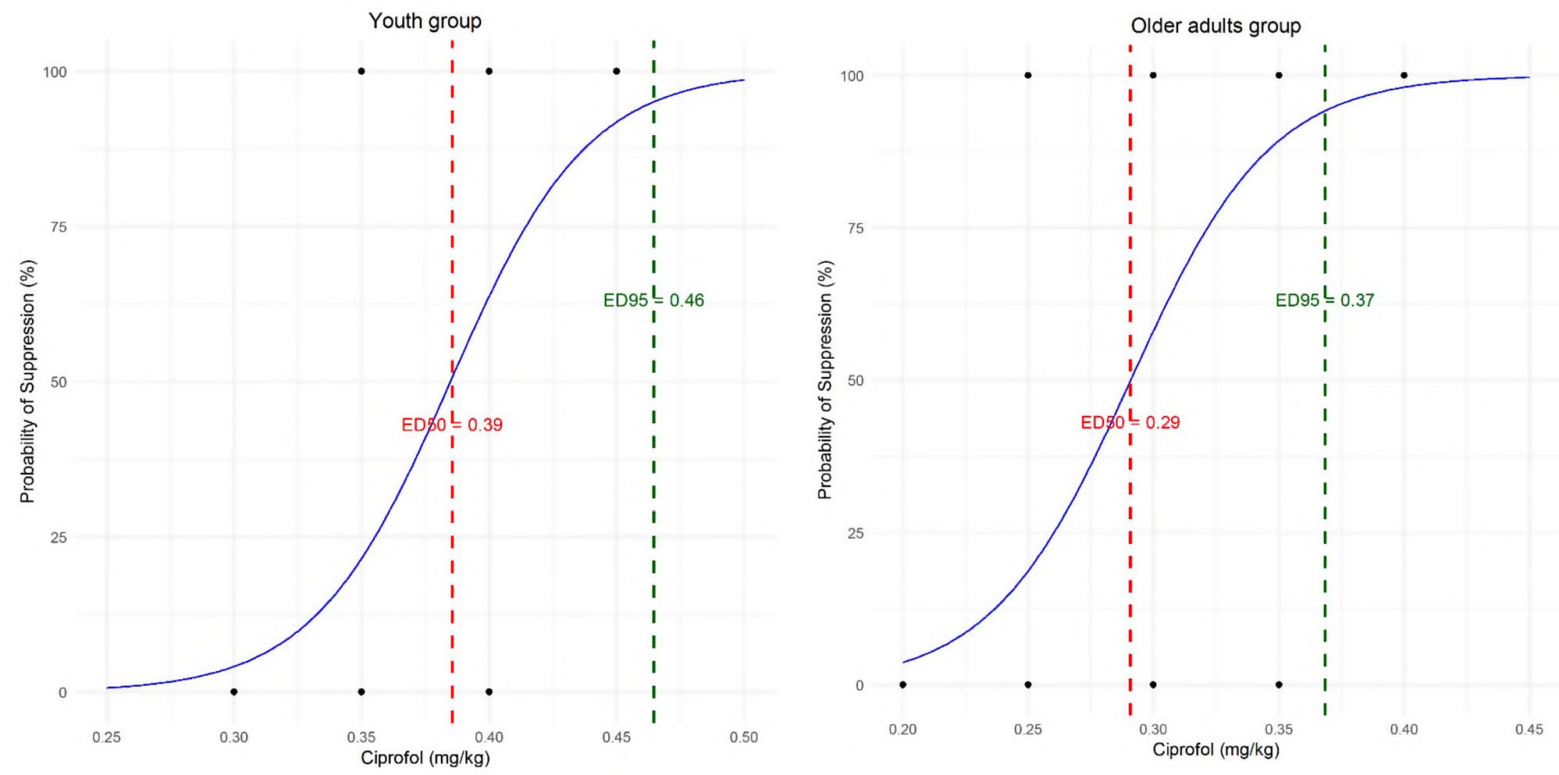
Dose-response curves for the Youth group (left) and the Older adult group (right); The red line represents ED_50_, while the green line represents ED_95_

In the youth group, the ED_50_ and ED_95_ of ciprofol for suppressing the LMA insertion response were 0.38 mg·kg^− 1^ (95% CI, 0.35–0.41) and 0.46 mg·kg^− 1^ (95%CI, 0.40–0.56), respectively. In the older adult group, the respective ED_50_ and ED_95_ were 0.29 mg·kg^− 1^ (95% CI, 0.26–0.32) and 0.37 mg·kg^− 1^ (95% CI, 0.30–0.78). The Dixon up-and-down plots for both the older adult and youth groups are shown in Fig. 2. The dose-response curves for the groups are shown in Fig. 3.

## Secondary outcomes

**Fig. 4:**
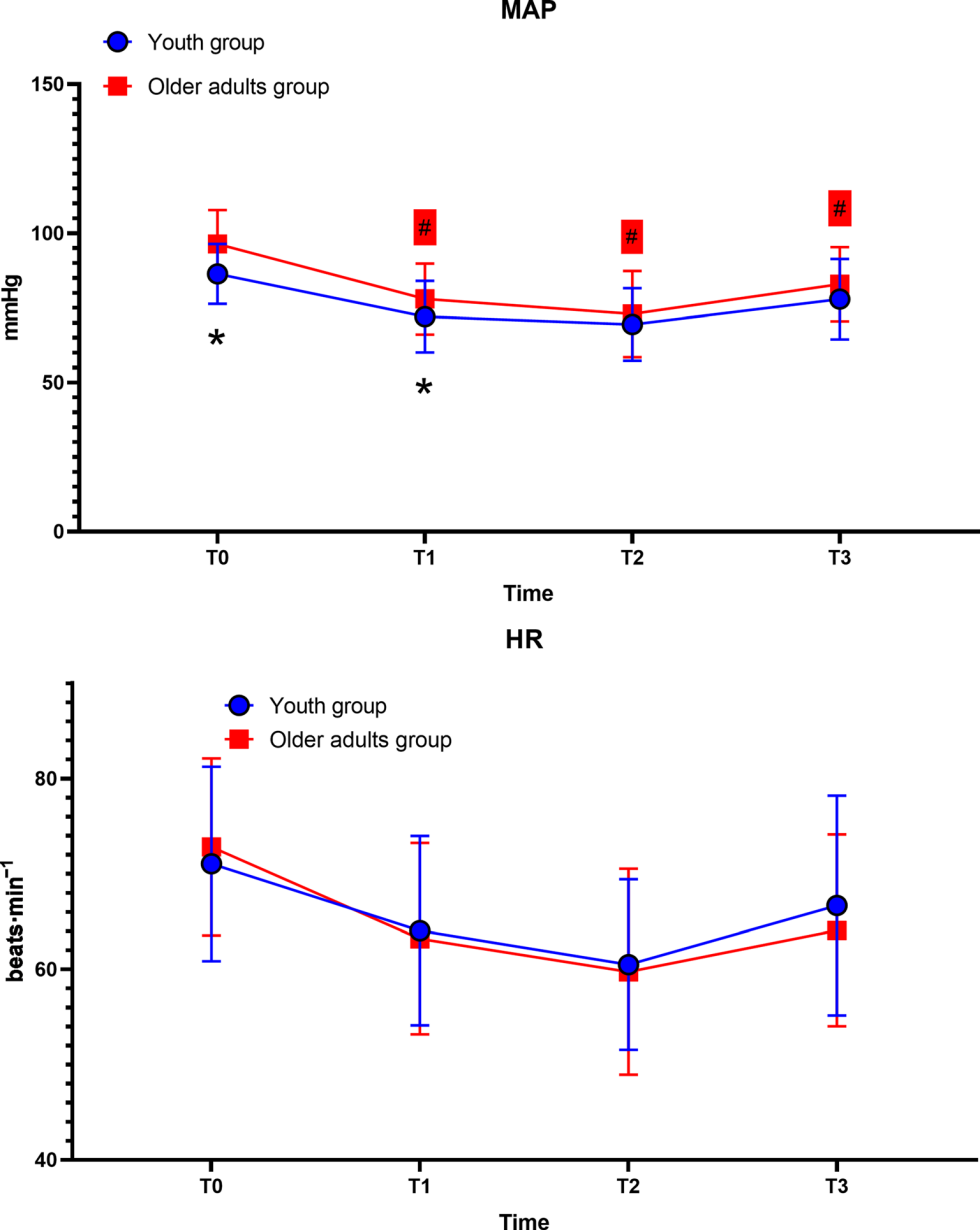
MAP Mean Arterial Pressure; HR Heart Rate; # *P* < 0.05 within-group comparisons; * *P* < 0.05 between-group comparisons

In the older adults group, the mean arterial pressure (MAP) at baseline (T0) was significantly lower than at T1 and T2, with no other statistical differences observed. In the youth group, neither heart rate nor MAP showed statistically significant changes over time. At baseline (T0) and T1, the MAP in the older adults group was significantly higher than in the youth group, while no other statistical differences were noted (Fig. 4).

Incidence of Adverse Events (Table 2).

**Table 2:** Incidence of Adverse Events

	Youth group (*n* = 22)	Older adults group (*n* = 24)	*P* value
Injection pain	1(4.54%)	0(0)	0.96
Hypotension	5(22.73%)	9(37.50%)	0.44
Bradycardia	1(4.55%)	4(16.67%)	0.40
Time of laryngeal mask placement	36.36 ± 9.88	37.33 ± 9.35	0.73

Data are presented as the mean ± SD or the number M Male; P-value: Independent Samples t-Test/Chi-Square Test

Regarding adverse reactions, the incidence of injection pain in the youth group was 4.54%, higher than 0% in the older adult group. The incidence of hypotension, defined as a 30% reduction in mean arterial pressure from the baseline [26], was 37.50% in the older adult group, higher than 22.73% in the youth group. Similarly, the incidence of bradycardia was also higher in the older adult group at 16.67%, compared to 4.55% in the youth group. Despite these findings, no statistically significant differences were observed in the incidences of injection pain, bradycardia, hypotension, and LMA insertion time between the two groups. Additionally, no other types of adverse reactions were identified in the study.

## Discussion

This study aimed to assess the ED_50_ of ciprofol for suppressing LMA insertion reactions in young and older adult patients. The results indicated that the ED_50_ of ciprofol for inhibiting the LMA insertion reaction in young and older adult patients was 0.38 mg·kg^− 1^ (95% CI, 0.35–0.41) and 0.29 mg·kg^− 1^ (95% CI, 0.26–0.32), respectively. The 95% confidence intervals between the two groups did not overlap, showing a statistically significant difference. Therefore, clinical practice necessitates tailored ciprofol dosing for young and older adult patients.

In our study, the ED_50_ of ciprofol combined with Sufentanil for suppressing LMA insertion reactions in young and older adult patients was 0.38 mg·kg^− 1^ (95% CI, 0.35–0.41) and 0.29 mg·kg^− 1^ (95% CI, 0.26–0.32), respectively, with no overlapping confidence intervals and statistically significant differences, revealing the variability in ciprofol requirements across different age groups, particularly higher needs in younger patients compared to the older adult. This phenomenon may be attributed to structural and functional changes in various physiological systems with age, including the brain, heart, liver, kidneys, and body composition, which make older adult patients more sensitive to anesthetic drugs and circulatory fluctuations [27–29]. Therefore, in clinical practice, anesthetic management of older adult patients requires more precision and caution, especially in the use of anesthetic drugs like ciprofol, necessitating lower dosages compared to younger patients. During the study, one patient in the youth group and two patients in the older adults group had unsuccessful LMA insertions due to inadequate sealing. Following the study protocol, these patients subsequently underwent tracheal intubation, which did not lead to any further adverse reactions.

While this research provides insights into ciprofol’s application for LMA insertion in both older adults and young patients, it has certain limitations. The study cohorts differed in ASA classifications, with older adults often presenting a more complex health profile due to comorbidities, potentially affecting anesthetic response. Although older adults had a higher incidence of adverse effects like injection pain, bradycardia, and hypotension compared to younger patients (Injection pain: 4.17% vs. 9.09%, *P* = 0.94; Hypotension: 37.50% vs. 22.73%, *P* = 0.44; Bradycardia: 16.67% vs. 4.55%, *P* = 0.40), these differences were not statistically significant, likely due to the limited sample size. This highlights the need for larger studies to better define age-related differences in response to ciprofol. Furthermore, the restriction to patients with ASA grades 1–3 may limit the broader applicability of these findings. Future studies should include a wider range of ASA classifications to better understand ciprofol’s effects across diverse patient profiles.

## Conclusion

In this study, ED_50_ for inhibiting LMA insertion responses with propofol in the young group and the older adult group were determined to be 0.38 mg·kg^− 1^ (95% CI, 0.35–0.41) and 0.29 mg·kg^− 1^ (95% CI, 0.26–0.32), respectively. The non-overlapping intervals indicate a statistically significant difference between these two age groups. This finding suggests that there are variations in ciprofol dosing requirements across different age groups, highlighting the importance of considering age when adjusting dosages in anesthetic practice.

## Electronic Supplementary Material

Below is the link to the electronic supplementary material.

Supplementary Material 1

Supplementary Material 2

Supplementary Material 3

Supplementary Material 4

## Data Availability

The data used in this study are included in the manuscript. For further inquiries, please contact the corresponding author.

## Abbreviations

LMA: Laryngeal mask airway
ED_50_: Median effective dose
ED_95_: Effective Dose 95
BP: Blood pressure
HR: Heart rate
SpO_2_: Pulse oximetry
MOAA/S: Modified Observer’s Alertness/Sedation scale
