# Supplementary figures and images for: The median effective dose of ciprofol combined with sufentanil in suppressing the laryngeal mask airway insertion response in both young and older adult patients
Source: BMC Anesthesiol. 2024 Dec 19;24:464. doi: 10.1186/s12871-024-02855-5 (PMC11658249; doi:10.1186/s12871-024-02855-5)

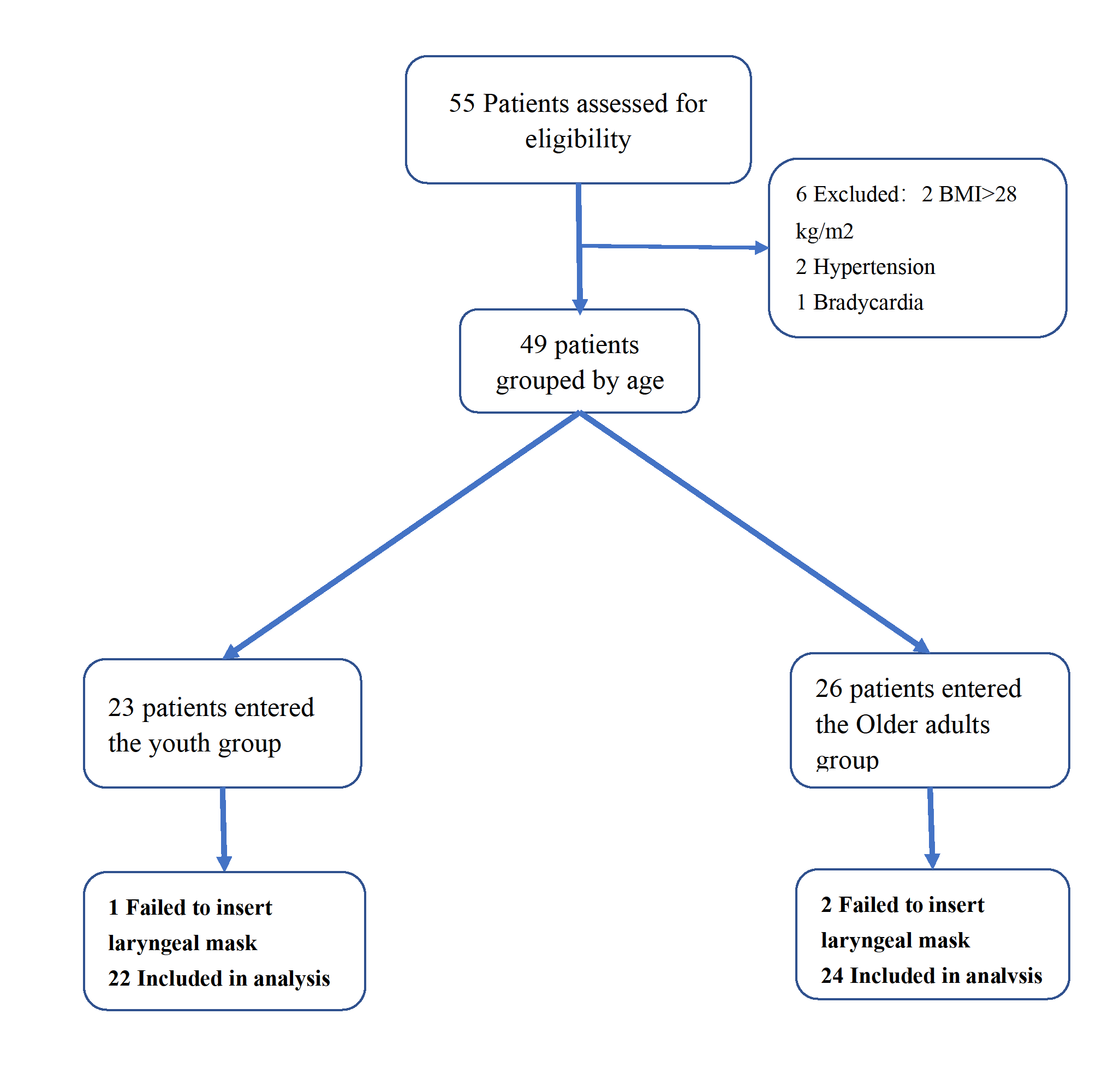

Supplement: Supplementary file 1 — Supplementary Material 1 [file 12871_2024_2855_MOESM1_ESM.tif]

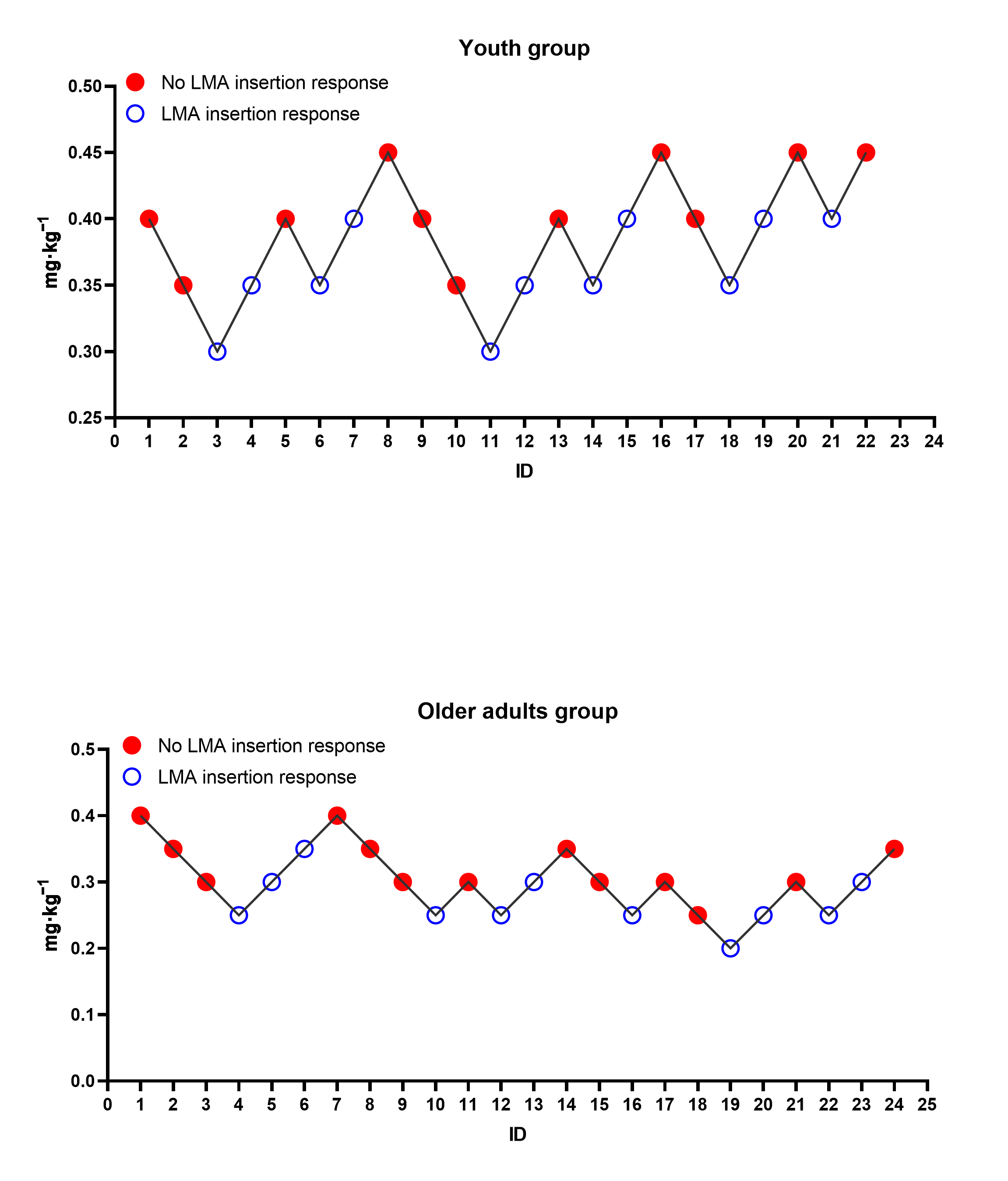

Supplement: Supplementary file 2 — Supplementary Material 2 [file 12871_2024_2855_MOESM2_ESM.tif]

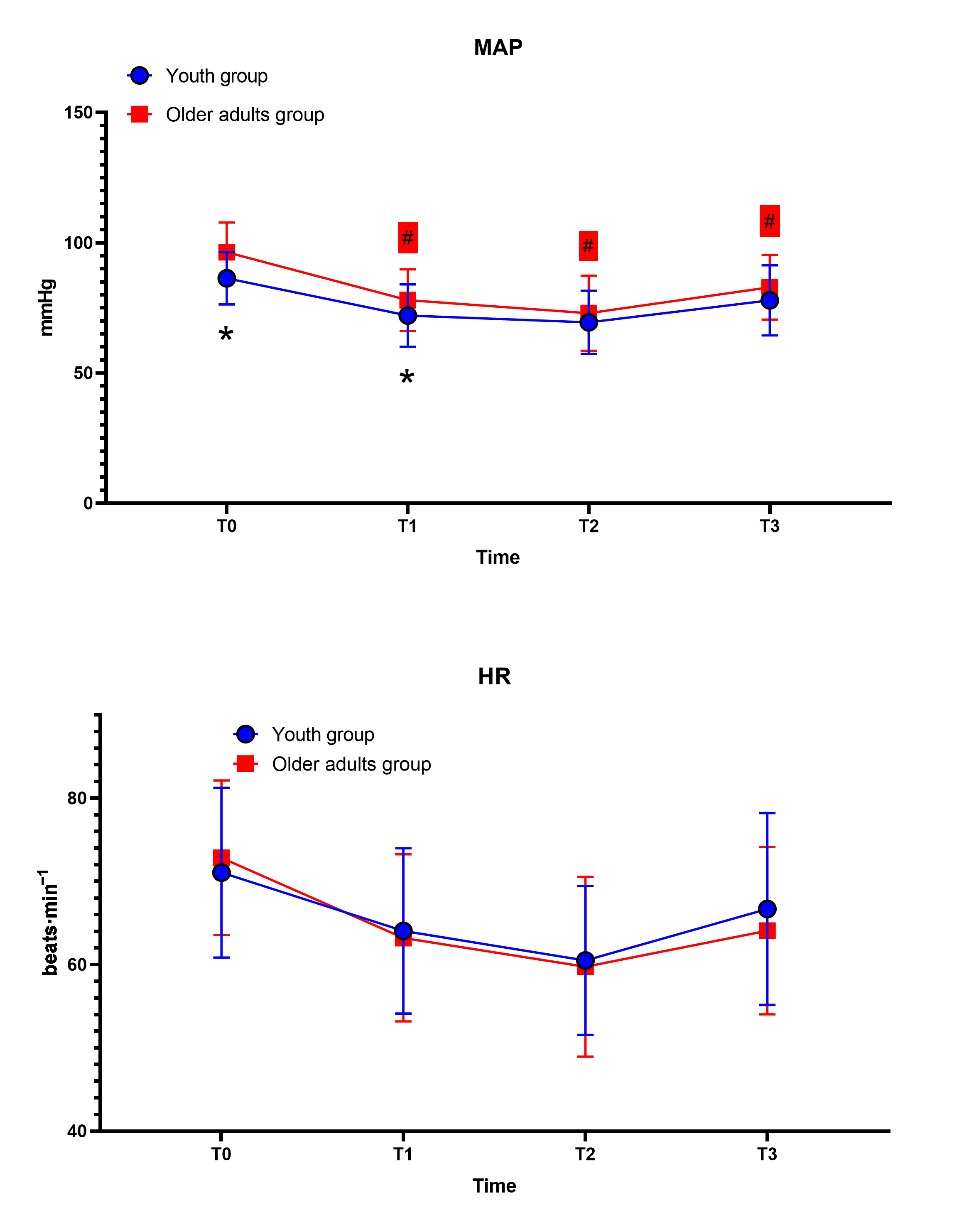

Supplement: Supplementary file 3 — Supplementary Material 3 [file 12871_2024_2855_MOESM3_ESM.tif]

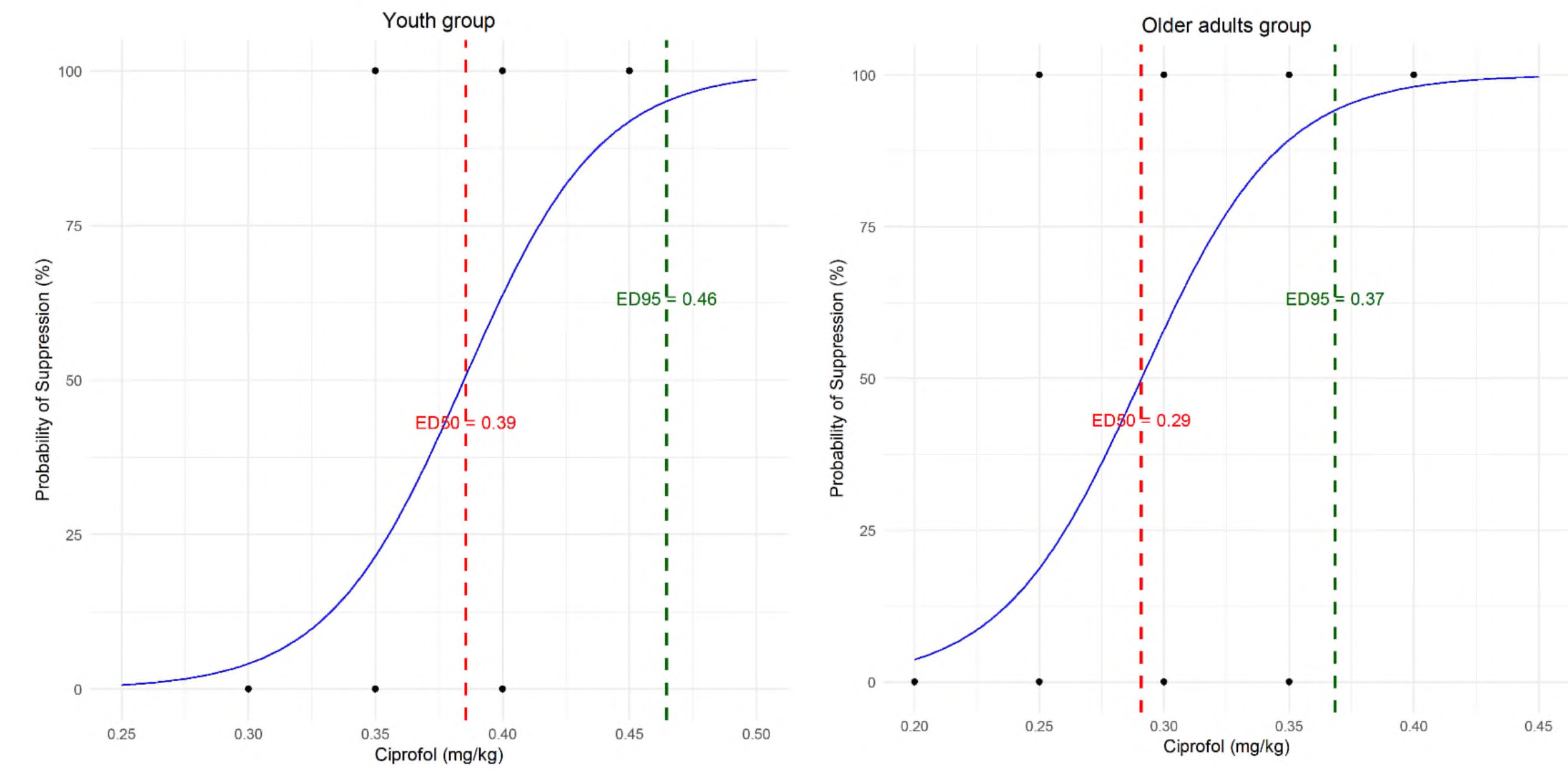

Supplement: Supplementary file 4 — Supplementary Material 4 [file 12871_2024_2855_MOESM4_ESM.tif]
